# MRI-based morphological and spatial characteristics of leptomeningeal metastasis: prognostic value in non-small cell lung cancer

**DOI:** 10.3389/fonc.2026.1764407

**Published:** 2026-04-10

**Authors:** Xin Chen, Xueying He, Tianli Zhang, Huiying Li, Cen Chen, Mingmin Huang, Aibin Guo, Tingting Yu, Yu Xie, Yongjuan Lin, Pengpeng Gui, Yue Wang, Mingyang He, Zhenyu Yin, Xue Liang

**Affiliations:** 1 Geriatrics, Nanjing Drum Tower Hospital Clinical College of Nanjing University of Chinese Medicine, Nanjing, China; 2 Department of Radiology, Affiliated Nanjing Drum Tower Hospital of Nanjing University Medical School, Nanjing, China; 3 Department of Geriatric Oncology, Affiliated Nanjing Drum Tower Hospital of Nanjing University Medical School, Nanjing, China

**Keywords:** leptomeningeal metastasis, magnetic resonance imaging black bloodsequence, morphological subtyping, NSCLC, prognostic factors, spatial distribution

## Abstract

**Background:**

Leptomeningeal metastasis (LM) represents a devastating complication of non-small cell lung cancer (NSCLC), with limited survival and poorly defined imaging-based prognostic markers.

**Purpose:**

This study evaluated the combined prognostic value of MRI-based morphological and spatial patterns in NSCLC patients with LM, whose prognostic relevance remains poorly defined.

**Methods:**

We retrospectively reviewed 71 NSCLC patients with LM confirmed by 3.0T black-blood MRI, selected from 109 initially screened after applying exclusion criteria. Patients were classified into linear (n=41) and mixed (n=30) morphological subtypes based on MRI, and stratified by the number of involved brain regions (>3 vs. ≤3). Clinical, imaging, and survival data were analyzed using Kaplan-Meier estimates and multivariate Cox regression to identify independent prognostic factors.

**Results:**

The mixed subtype exhibited a significantly higher lesion burden than the linear subtype (26.86 ± 26.72 vs. 7.25 ± 10.95, p<0.001). Involvement of more than three brain regions was associated with significantly shorter median overall survival (14 vs. 24 months, p=0.016). Multivariate analysis identified several independent adverse prognostic factors: increased lesion number (HR = 1.02, 95% CI: 1.01–1.03, p<0.01), temporal lobe invasion (HR = 1.96, 95% CI: 1.07-3.58, p=0.029), Regionsinvolved (HR = 0.52, p=0.020).

**Conclusion:**

MRI-based morphological subtyping and spatial distribution provide significant prognostic value in NSCLC-LM. The mixed morphology, extensive brain involvement (>3 regions), and invasions of specific location such as the tempoarl lobe is associated with poorer survival outcomes. These findings support the use of MRI phenotyping for risk-adapted clinical management in NSCLC-LM.

## 1 Introduction

NSCLC represents the predominant histological type of lung cancer, accounting for approximately 85% of all lung cancer cases. As a malignancy characterized by high global incidence and mortality rates, recent progress in targeted therapy and immunotherapy has substantially improved patient survival outcomes. Consequently, this has led to a rising incidence of brain metastases, which develop in 23% to 36% of advanced-stage cases (1–3). Brain metastases represent the most common type of intracranial metastatic tumor and are generally associated with a poor prognosis (4). The treatment of LM in NSCLC faces significant blood-brain barrier penetration challenges. Drug distribution in cerebrospinal fluid(CSF) exhibits marked heterogeneity, and the concentration gradient between CSF and brain parenchyma makes it difficult to achieve therapeutic concentrations across all lesions. Once LM occurs, tumor cells disseminate within the CSF spaces, leading to severe symptoms such as blindness, paralysis, and mental disorders, which significantly impair patients’ quality of life, with a median survival of approximately 4.5 months, posing a major clinical difficulty (5, 6). Existing studies suggest that survival outcome in lung cancer patients with LM can be influenced by different targeted therapies, intrathecal chemotherapy strategies, and genetic mutation profiles (5). Targeted therapy has significantly improved the survival prognosis of patients with leptomeningeal metastasis from NSCLC. Studies demonstrate that patients treated with tyrosine kinase inhibitors achieved a median progression-free survival of 6.9 months and a median overall survival of 15.0 months (7). For specific patient populations such as those with EGFR mutations, intrathecal chemotherapy as a local treatment modality may offer long-term benefits (8).

CSF analysis serves as a crucial method for diagnosing central nervous system infections, inflammation, and tumors, yet its sensitivity has limitations. Negative CSF test results may stem from the following factors: low pathogen load, inappropriate sampling timing, or localized lesions (such as tumors or dissections) failing to release sufficient biomarkers into the CSF. However, many prognostic factors remain unclear. Although CSF is the gold standard for confirming LM (9), magnetic resonance imaging (MRI) serves as a non-invasive imaging method with anirreplaceable role in its diagnosis and evaluation. When clinical manifestations strongly suggest central nervous system pathology despite negative CSF findings, black-blood sequence imaging can provide supplementary diagnostic confirmation (10, 11). Black-blood imaging is an advanced MRI technique that employs diffusion gradient fields to suppress signals from flowing blood, thereby presenting them as hypointense regions, while simultaneously attenuating signals from water molecules undergoing diffusion. Notably, the implementation of black-blood sequences has markedly enhanced the detection sensitivity of leptomeningeal and cerebellar metastatic lesions, with certain studies demonstrating sensitivity rates surpassing 90%—a significant improvement compared to conventional T1-weighted imaging sequences (12). As a result, MRI is increasingly important in the clinical diagnosis, treatment response assessment, and follow-up of LM. In recent years, the impact of spatial distribution patterns of brain metastases on prognosis has attracted growing interest. Beyond parenchymal brain metastases, studies have shown that the spatial and morphological characteristics and extent of LM are also closely related to patient outcome (13). For example, in breast cancer, different morphological subtypes of dural metastases demonstrate distinct clinical courses and prognostic features (14, 15). However, the relationship between MRI-based morphological subtypes, spatial distribution, and patient prognosis in NSCLC with LM remains insufficiently studied.

Based on clinical data, we hypothesize that the enhancement patterns and extent of LM may hold significant predictive value for prognosis, with more extensive involvement likely indicating poorer outcomes. This study utilizes 3D black-blood MRI sequences to systematically investigate the association between morphological characteristics and lesion extent in LM among patients with NSCLC. These imaging features are rigorously analyzed in conjunction with clinical parameters and survival outcomes. The results are anticipated to provide critical imaging evidence that could inform the development of personalized clinical management approaches for LM in NSCLC patients.

## 2 Materials and methods

### 2.1 Participants

This study enrolled NSCLC patients with LM who were treated at the Nanjing Drum Tower Hospital The Affiliated Hospital of Nanjing University Medical School from January 2023 to June 2024. The study was approved by the Ethics Committee of the Nanjing Drum Tower Hospital The Affiliated Hospital of Nanjing University Medical School (Ethics Approval No. 2023-LCYJ-MS-14), and written informed consent was obtained from all participants or their family members.

The diagnosis of LM was established according to the following criteria: (1) pathological confirmation of primary lung cancer with prior standardized treatment; (2) recent onset of neurological symptoms or signs; (3) detection of malignant cells in CSF cytology; and (4) typical MRI findings of the brain. A definitive diagnosis required fulfillment of criterion (1) plus at least two of criteria (2) to (4).

Inclusion criteria were: (1) age 18–75 years; (2) pathological diagnosis of lung adenocarcinoma meeting the above LM diagnostic criteria; (3) absence of significant bleeding symptoms, coagulopathy, or thrombotic diseases; (4) no severe cardiac, hepatic, or renal dysfunction. Exclusion criteria included: (1) incomplete baseline data or lost to follow-up; (2) clinically diagnosis of LM without MRI black blood sequence examination; and (3) poor image quality due to patient motion or other artifacts rendering MRI scans uninterpretable.

### 2.2 Clinical indicators

Clinical indicators in this study included overall survival (OS), age, gender, Karnofsky Performance Status (KPS), which is a standardized scale ranging from 0 to 100 assessing functional status in cancer patients, originally introduced by Karnofsky et al., 1948 (16), targeted drugs, anti-angiogenic therapy, intrathecal injection treatment protocols and the interval from initial diagnosis to LM diagnosis. Laboratory indicators comprised CSF cytology results for gene mutations and mutation types (EGFR, ALK, HER2, and TP53). Imaging indicators encompassed the location of lesions, the number of lesions, and the morphology of lesions.

### 2.3 MRI acquisition protocol

All MRl examinations were conducted on a uMR790^®^ 3.0T MR system (Shangha United lmaging Healthcare Co., Ltd.) scanner with a 32-channel head coil. Conventional T1-weighted gradient recalled echo (GRE) sequences were applied with a scanning range covering the whole brain. The contrast agent gadobutrol (Gd-DTPA) was injected via the elbow vein using a high-pressure syringe at a dose of 0.12 mmol/kg and an injection rate of 3–5 mL/s. Within 5 minutes after contrast injection, the 3D-T1 GRE and 3D-MATRlX sequences were acquired.

### 2.4 MRI image assessment

MRI images were independently evaluated by two neuroradiologists, Two physicians (with 11 and 8 years of experience in neural MRI respectively) conducted qualitative classification of enhancement pattern continuity, thickness uniformity, and nodule presence based on the high-contrast resolution of 3D-MATRIX sequences through visual assessment. Linear enhancement was defined as diffuse continuous thin linear enhancement; mixed pattern was defined as linear enhancement with focal nodules in the background. Although quantitative criteria based on specific enhancement intensity thresholds have not been established, the inter-rater agreement was good. Any discrepancies in assessment were resolved through consensus discussion between the two physicians.

### 2.5 Grouping criteria

**Figure 1:**
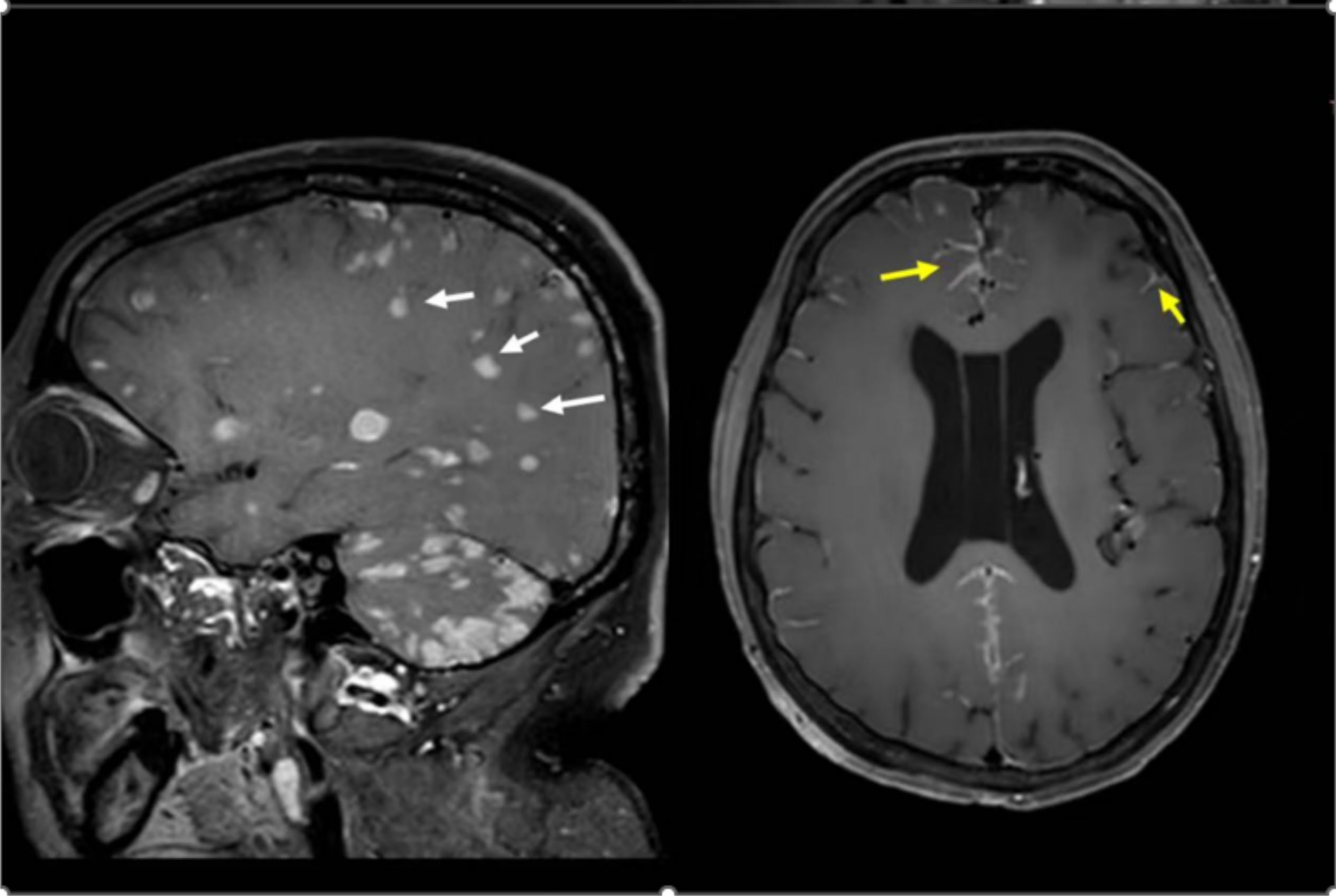
The imaging enhancement patterns of LM. The lesion indicated by the white arrow in the left image is of nodular type, while the lesion marked by the yellow arrow in the right image demonstrates a linear pattern.

LM was classified into three morphological subtypes: linear type (continuous linear or patchy enhancement on the surface of sulci, cisterns, or ependyma), nodular type (focal nodular enhancement on the surface of the brain/spinal cord, cranial nerve roots, or subependymal regions), and mixed type (coexistence of both linear and nodular enhancement patterns) (**Figure 1**).

### 2.6 Outcome evaluation

OS was defined as the duration from the date of LM diagnosis to death from any cause or the last follow-up. Patients underwent monthly MRI examinations following NSCLC-LM diagnosis. The primary endpoint was OS, and the secondary endpoint was KPS.

### 2.7 Statistical analysis

All statistical analyses were performed using R software (version 4.5.1). Continuous variables were expressed as mean ± standard deviation (SD) or median (interquartile range), as appropriate, and compared using the independent samples t-test or Mann–Whitney U test. Categorical variables were summarized as frequencies (percentages) and analyzed using the χ² test or Fisher’s exact test.Overall survival (OS) was defined as the time from the date of LM diagnosis to death from any cause or last follow-up. Survival curves were estimated using the Kaplan–Meier method and compared using the log-rank test.To identify independent prognostic factors, Cox proportional hazards regression models were constructed. Variables with p < 0.10 in univariate analysis or considered clinically relevant were included in multivariate models. Hazard ratios (HRs) with 95% confidence intervals (CIs) were reported.Model assumptions and performance were evaluated to ensure robustness. The proportional hazards assumption was assessed, and multicollinearity among predictors was examined before multivariate modeling.

## 3 Results

### 3.1 Patient baseline characteristic of mixed and linear

**Figure 2:**
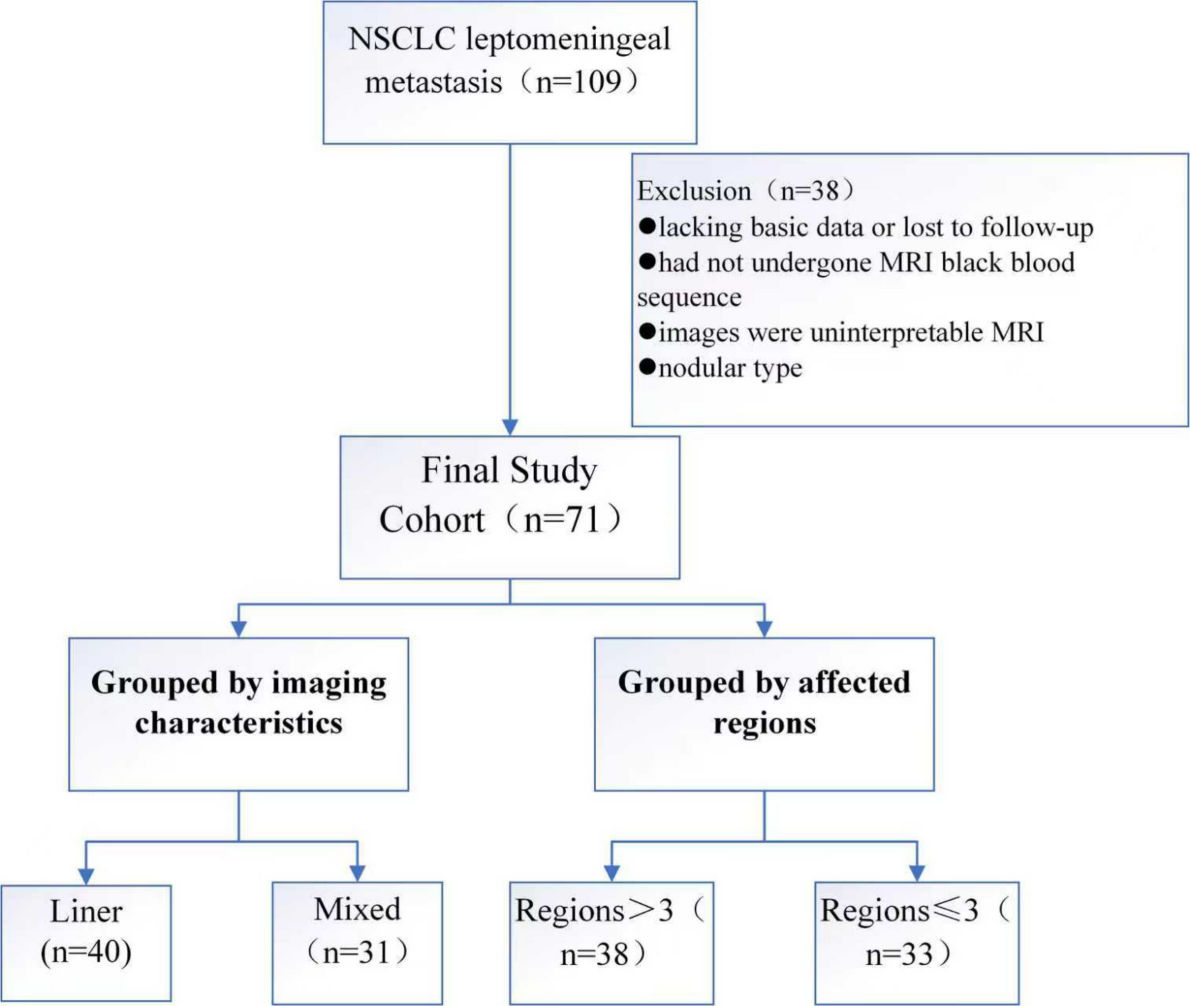
Flow chart of patient enrollment. NSCLC.

A total of 109 patients were initially included in this study. After excluding 8 patients due to missing baseline data or loss to follow-up, 12 patients clinically diagnosed with LM but without black blood sequence examination, 15 patients with unrecognizable MRI images, and 3 patients with nodular imaging features, 71 LM patients were finally analyzed (**Figure 2**). The cohort consisted of 31 males (43.66%) and 40 females (56.34%), with ages ranging from 37 to 75 years (mean age 56 ± 8.19 years). According to imaging enhancement features, 41 (57.75%) cases were classified as linear type and 30 (42.25%) as mixed type. Due to limited number of nodular type cases (n = 3), only linear and mixed types were compared for statistical robustness (**Table 1**).

**Table 1:** Clinical characteristics of LM patients grouped by imaging features.

Variables	Linear type (n = 41)	Mixed type (n = 30)	Statistic	*P*
Left lateral ventricle, Mean ± SD	18.40 ± 9.44	17.77 ± 9.87	t=0.27	0.787
Right Lateral Ventricle, Mean ± SD	15.85 ± 8.92	15.61 ± 9.17	t=0.11	0.914
Detection Age, Mean ± SD	55.93 ± 12.82	53.28 ± 7.26	t=1.00	0.319
Development Time, Mean ± SD	33.24 ± 23.05	33.62 ± 36.42	t=-0.05	0.958
KPS, Mean ± SD	61.95 ± 12.09	62.67 ± 12.58	t=-0.24	0.809
Os, Mean ± SD	22.15 ± 14.55	17.50 ± 12.81	t=1.40	0.167
Lesions number, Mean ± SD	7.25 ± 10.95	26.86 ± 26.72	t=-3.73	**<0.001**
Age, M (Q_1_, Q_3_)	58.00 (54.00, 66.00)	54.00 (52.00, 59.00)	Z=-2.26	**0.024**
Sex, n(%)			χ²=0.19	0.662
female	24 (58.54)	16 (53.33)		
male	17 (41.46)	14 (46.67)		
EGFR, n(%)			χ²=0.00	0.988
no	11 (26.83)	8 (26.67)		
yes	30 (73.17)	22 (73.33)		
ALK, n(%)			χ²=0.72	0.395
no	38 (92.68)	25 (83.33)		
yes	3 (7.32)	5 (16.67)		
HER2, n(%)			χ²=5.03	**0.025**
no	41 (100.00)	25 (83.33)		
yes	0 (0.00)	5 (16.67)		
Tp53, n(%)			χ²=1.70	0.193
no	40 (97.56)	26 (86.67)		
yes	1 (2.44)	4 (13.33)		
Ventricular Dilatation, n(%)			χ²=0.00	0.947
no	29 (70.73)	21 (70.00)		
yes	12 (29.27)	9 (30.00)		
Brain Parenchyma, n(%)			χ²=9.46	**0.002**
no	30 (73.17)	11 (36.67)		
yes	11 (26.83)	19 (63.33)		
CSF, n(%)			χ²=1.15	0.285
no	9 (21.95)	10 (33.33)		
yes	32 (78.05)	20 (66.67)		
Frontal, n(%)			χ²=11.55	**<0.001**
no	29 (70.73)	9 (30.00)		
yes	12 (29.27)	21 (70.00)		
Parietal, n(%)			χ²=0.28	0.595
no	19 (46.34)	12 (40.00)		
yes	22 (53.66)	18 (60.00)		
Temporal, n(%)			χ²=1.66	0.197
no	17 (41.46)	8 (26.67)		
yes	24 (58.54)	22 (73.33)		
Occipital, n(%)			χ²=3.86	**0.049**
no	12 (29.27)	3 (10.00)		
yes	29 (70.73)	27 (90.00)		
Cerebellum, n(%)			χ²=4.42	**0.035**
no	18 (43.90)	6 (20.00)		
yes	23 (56.10)	24 (80.00)		
Brainstem, n(%)			χ²=5.93	**0.015**
no	27 (65.85)	11 (36.67)		
yes	14 (34.15)	19 (63.33)		
Regions involved, n(%)			χ²=3.61	0.057
>3	18 (43.90)	20 (66.67)		
≤3	23 (56.10)	10 (33.33)		
Targeted Therapy, n(%)			χ²=1.13	0.288
dual third-generation drugs	23 (56.10)	13 (43.33)		
others	18 (43.90)	17 (56.67)		
EFGR, n(%)			χ²=3.95	0.139
19del	12 (29.27)	4 (13.33)		
21L858R	18 (43.90)	12 (40.00)		
Others	11 (26.83)	14 (46.67)		
intrathecal chemotherapy(mg), n(%)			χ²=0.60	0.438
Pemetrexed**<**30	13 (31.71)	7 (23.33)		
Pemetrexed≥30	28 (68.29)	23 (76.67)		
Anti-angiogenic therapy, n(%)			χ²=0.51	0.475
Bevacizumba	17 (41.46)	15 (50.00)		
Others	24 (58.54)	15 (50.00)		

SD, standard deviation; KPS, karnofsky performance status;OS, overall survival;EGFR, ​epidermal growth factor receptor; ALK, anaplastic lymphoma kinase; HER2, human epidermal growth factor receptor 2; TP53, tumor protein 53;CSF. Bold values indicate statistically significant differences (p < 0.05).

### 3.2 Differences between mixed and linear groups

The mixed type demonstrated a significantly higher average number of lesions compared to the linear type (26.86 ± 26.72 vs. 7.25 ± 10.95, p < 0.05). HER2 mutation was only observed in the mixed type, while no significant differences were found in other gene mutation types (EGFR and TP53) between the groups. In terms of lesion spatial distribution, the mixed type showed significantly higher involvement in the frontal lobe, cerebellum, and brainstem (p < 0.05, **Table 1**).

### 3.3 Survival analysis of mixed and linear groups

**Figure 3:**
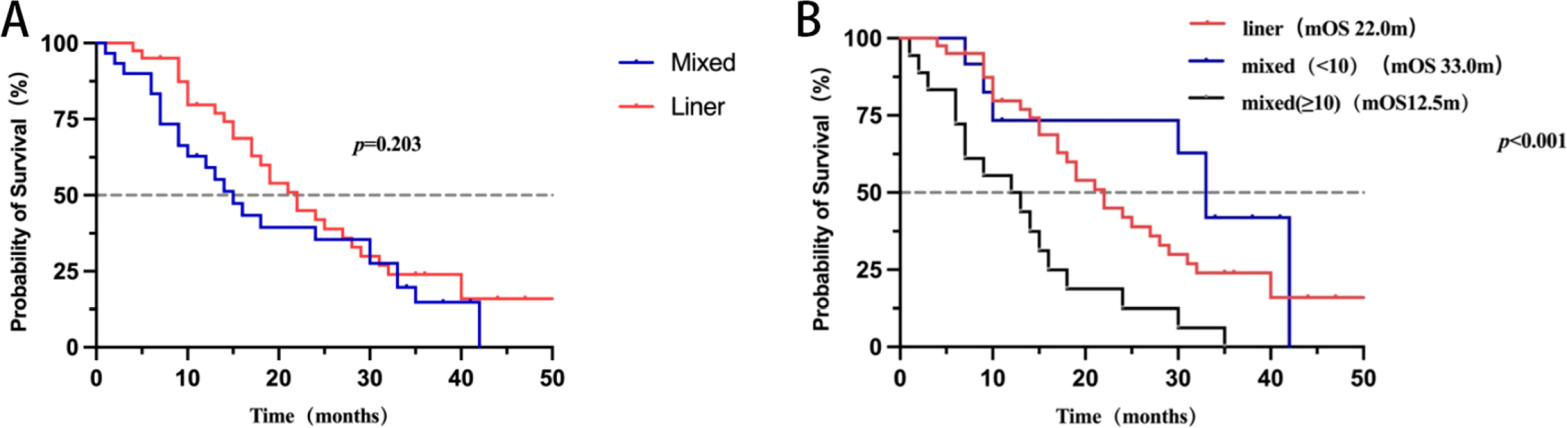
**(A)** Overall survival grouped by MRI features; **(B)** Overall survival grouped by MRI characteristics and lesion count.

**Figure 4:**
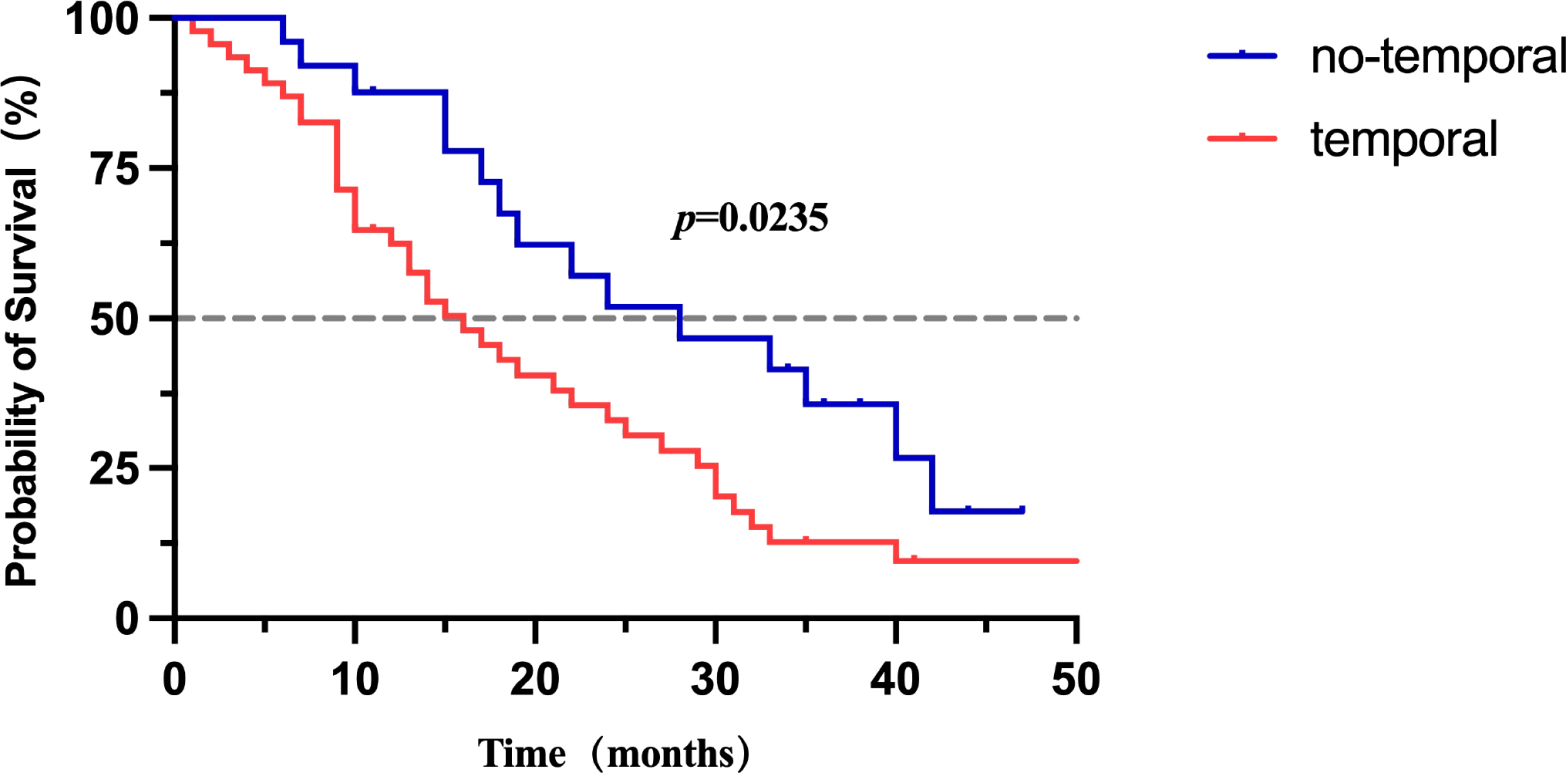
Overall survival grouped by temporal lobe involvement.

While no statistically significant difference in median overall survival (mOS) was detected between the linear and mixed types (Log-rank test, P > 0.05), the mixed type exhibited a tendency toward poorer survival outcomes (**Figure 3A**; **Supplementary Table 1**). Notably, the “mixed-type group” (i.e., the group containing both ≥10 and <10 nodules) showed significant prognostic differences (Logrank P<0.001), indicating that the number of nodules has a significant impact on survival rates(**Figure 3B**).Additionally, temporal lobe involvement was associated with significantly lower mOS (P < 0.05, **Figure 4**; **Supplementary Table 2**).

### 3.4 Survival analysis of therapy

**Figure 5:**
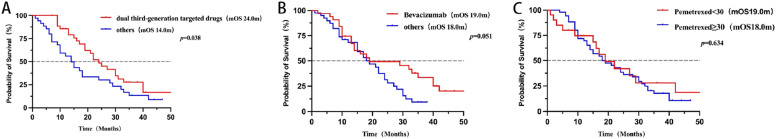
**(A)** Overall survival grouped by Targeted durgs; a**(B)** Overall survival grouped by Anti-angiogenic therapy; **(C)** Overall survival grouped by Intrathecal injection dose.

Patients receiving dual third-generation targeted drugs demonstrated significantly superior mOS compared to those on other medications (p<0.05, **Figure 5A**). Although the difference did not reach statistical significance, bevacizumab showed a trend toward improved survival (p=0.051, **Figure 5B**). However, when pemetrexed disodium was administered via intrathecal injection, no significant difference in survival rates was observed between the two dosage groups (p=0.634, **Figure 5C**).

### 3.5 Cox regression analysis

To further evaluate the impact of imaging features on overall survival, Cox proportional hazards regression analysis was performed (**Table 2**). In the univariate Cox analysis, lesion number (HR = 1.07, 95% CI: 1.02–1.11, p = 0.002) was significantly associated with shorter OS. Frontal lobe involvement (HR = 1.71, 95% CI: 1.00–2.92, p = 0.052) and brainstem involvement (HR = 1.65, 95% CI: 0.96–2.84, p = 0.069) demonstrated a marginal association with poorer survival. HER2 positivity also showed a trend toward adverse prognosis (HR = 2.23, 95% CI: 0.88–5.64, p = 0.091).

**Table 2:** Results of multivariate cox regression analysis for imaging predictors.

Variables	Univariate analysis					Multivariate analysis				
	β	S.E	Z	*P*	HR (95%CI)	β	S.E	Z	*P*	HR (95%CI)
HER2										
no					1.00 (Reference)					
yes	0.80	0.47	1.69	0.091	2.23 (0.88 ~ 5.64)					
Frontal										
no					1.00 (Reference)					
yes	0.53	0.27	1.95	0.052	1.71 (1.00 ~ 2.92)					
Occipital										
no					1.00 (Reference)					
yes	-0.09	0.32	-0.29	0.774	0.91 (0.49 ~ 1.71)					
Cerebellum										
no					1.00 (Reference)					
yes	0.33	0.29	1.14	0.255	1.39 (0.79 ~ 2.43)					
Brainstem										
no					1.00 (Reference)					
yes	0.17	0.28	0.63	0.528	1.19 (0.69 ~ 2.04)					
Lesions number	0.02	0.01	3.30	**<.001**	1.02 (1.01 ~ 1.03)	0.02	0.01	3.30	**<.001**	1.02 (1.01 ~ 1.03)
Age	0.01	0.02	0.35	0.729	1.01 (0.97 ~ 1.04)					
Brain Parenchyma										
no					1.00 (Reference)					
yes	0.50	0.28	1.82	0.069	1.65 (0.96 ~ 2.84)					

HR, Hazard Ratio; CI, Confidence Interval. Bold values indicate statistically significant differences (p < 0.05).

In the multivariate Cox regression model, after adjusting for potential confounders, lesion number remained an independent prognostic factor (HR = 1.06, 95% CI: 1.01–1.10, p = 0.011). Other variables, including frontal lobe involvement, cerebellar involvement, brainstem involvement, age, and brain parenchymal invasion, did not retain statistical significance in the adjusted model (p > 0.05).

### 3.6 Patient baseline characteristic of lesion spatial distribution

Patients were stratified into two groups based on whether the number of involved regions was >3 (n=38, 53.52%) or ≤3 (n=33, 46.48%). Significant differences were observed between the two groups in OS, number of lesions, number of involved brain regions, age, and CSF cytology (P < 0.05, **Table 3**).

**Table 3:** Clinical characteristics of LM patients grouped by the number of brain-involved regions.

Variables	>3 (n = 38)	≤3 (n = 33)	Statistic	*P*
DetectionAge, Mean ± SD	53.29 ± 7.25	56.66 ± 13.94	t=-1.23	0.224
DevelopmentTime, Mean ± SD	32.37 ± 21.03	34.62 ± 36.78	t=-0.32	0.749
KPS, Mean ± SD	63.16 ± 12.33	61.21 ± 12.19	t=0.67	0.507
Os, Mean ± SD	15.95 ± 12.27	25.06 ± 14.33	t=-2.89	**0.005**
Lesionsnumber, Mean ± SD	23.86 ± 25.46	5.81 ± 8.43	t=4.06	**<0.001**
Age, M (Q_1_, Q_3_)	54.00 (52.00, 59.00)	61.00 (55.00, 68.25)	Z=-2.75	**0.006**
Sex, n(%)			χ²=0.04	0.845
female	21 (55.26)	19 (57.58)		
male	17 (44.74)	14 (42.42)		
EGFG, n(%)			χ²=0.97	0.325
no	12 (31.58)	7 (21.21)		
yes	26 (68.42)	26 (78.79)		
ALK, n(%)			χ²=0.84	0.359
no	32 (84.21)	31 (93.94)		
yes	6 (15.79)	2 (6.06)		
HER2, n(%)			χ²=0.59	0.444
no	34 (89.47)	32 (96.97)		
yes	4 (10.53)	1 (3.03)		
Tp53, n(%)			χ²=0.03	0.870
no	36 (94.74)	30 (90.91)		
yes	2 (5.26)	3 (9.09)		
VentricularDilatation, n(%)			χ²=2.07	0.150
no	24 (63.16)	26 (78.79)		
yes	14 (36.84)	7 (21.21)		
BrainParenchyma, n(%)			χ²=2.01	0.156
no	19 (50.00)	22 (66.67)		
yes	19 (50.00)	11 (33.33)		
CSF, n(%)			χ²=5.02	**0.025**
no	6 (15.79)	13 (39.39)		
yes	32 (84.21)	20 (60.61)		
Frontal, n(%)			χ²=19.85	**<0.001**
no	11 (28.95)	27 (81.82)		
yes	27 (71.05)	6 (18.18)		
Parietal, n(%)			χ²=16.99	**<0.001**
no	8 (21.05)	23 (69.70)		
yes	30 (78.95)	10 (30.30)		
Temporal, n(%)			χ²=13.52	**<0.001**
no	6 (15.79)	19 (57.58)		
yes	32 (84.21)	14 (42.42)		
Occipital, n(%)			χ²=12.35	**<0.001**
no	2 (5.26)	13 (39.39)		
yes	36 (94.74)	20 (60.61)		
Cerebellum, n(%)			χ²=41.75	**<0.001**
no	0 (0.00)	24 (72.73)		
yes	38 (100.00)	9 (27.27)		
Brainstem, n(%)			χ²=19.85	**<0.001**
no	11 (28.95)	27 (81.82)		
yes	27 (71.05)	6 (18.18)		

Bold values indicate statistically significant differences (p < 0.05).

### 3.7 Differences between involved regions >3 and ≤3 groups

Subgroup analysis revealed significant differences in involvement of the frontal, parietal, temporal, occipital, cerebellum, and brainstem regions (P < 0.05, **Table 3**).

### 3.8 Survival analysis

**Figure 6:**
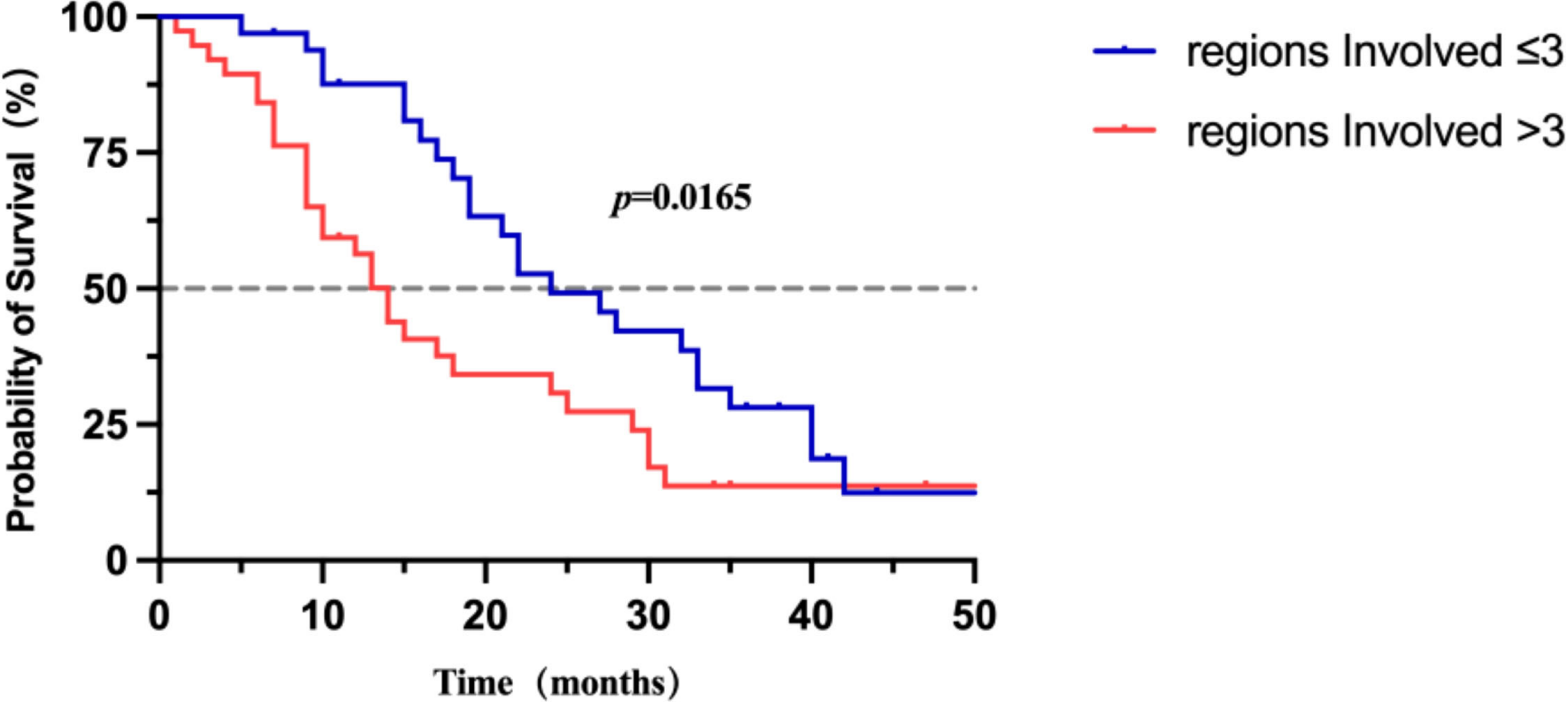
Overall survival grouped by number of affected regions.

Patients with ≤3 involved regions had significantly longer median survival compared to those with >3 involved regions (**Figure 6**; **Supplementary Table 3**).

### 3.9 Cox regression analysis

In the Cox regression analysis, univariate results showed that temporal lobe involvement was significantly associated with poorer OS (HR = 1.96, 95% CI: 1.07–3.58, p = 0.029). Patients with ≤3 involved regions exhibited a significantly reduced risk of death compared with those with >3 regions involved (HR = 0.52, 95% CI: 0.30–0.90, p = 0.020), indicating a protective effect of limited spatial dissemination. Other regional variables, including frontal, parietal, occipital, cerebellar, brainstem involvement, as well as CSF cytology and age, were not significantly associated with OS in the Cox model (p > 0.05).In the multivariate Cox regression model, temporal lobe involvement remained an independent adverse prognostic factor (HR = 1.96, 95% CI: 1.07–3.58, p = 0.029). The protective effect of ≤3 involved regions persisted, further confirming that extensive spatial dissemination (>3 regions) independently predicts worse survival outcomes. (**Table 4**).

**Table 4:** Results of multivariate cox regression analysis for number of brain-involved regions.

Variables	Univariate analysis					Multivariate analysis				
	β	S.E	Z	*P*	HR (95%CI)	β	S.E	Z	*P*	HR (95%CI)
CSF										
no					1.00 (Reference)					
yes	-0.01	0.31	-0.04	0.971	0.99 (0.54 ~ 1.82)					
Frontal										
no					1.00 (Reference)					
yes	0.53	0.27	1.95	0.052	1.71 (1.00 ~ 2.92)					
Parietal										
no					1.00 (Reference)					
yes	0.27	0.28	0.98	0.326	1.31 (0.76 ~ 2.26)					
Temporal										
no					1.00 (Reference)					1.00 (Reference)
yes	0.67	0.31	2.19	**0.029**	1.96 (1.07 ~ 3.58)	0.67	0.31	2.19	**0.029**	1.96 (1.07 ~ 3.58)
Occipital										
no					1.00 (Reference)					
yes	-0.09	0.32	-0.29	0.774	0.91 (0.49 ~ 1.71)					
Cerebellum										
no					1.00 (Reference)					
yes	0.33	0.29	1.14	0.255	1.39 (0.79 ~ 2.43)					
Brainstem										
no					1.00 (Reference)					
yes	0.17	0.28	0.63	0.528	1.19 (0.69 ~ 2.04)					
Age	0.01	0.02	0.35	0.729	1.01 (0.97 ~ 1.04)					
Regionsinvolved										
>3					1.00 (Reference)					
≤3	-0.64	0.28	-2.32	**0.020**	0.52 (0.30 ~ 0.90)					

HR, Hazard Ratio; CI, Confidence Interval. Bold values indicate statistically significant differences (p < 0.05).

## 4 Discussion

This study presents the first application of black blood sequence MRI in the evaluation of NSCLC patients with LM, providing a systematic analysis of imaging characteristics, spatial lesion distribution, and their prognostic implications. Patients with mixed imaging phenotypes demonstrated higher lesion burden and a tendency toward shorter mOS compared to those with linear type presentations. Notably, involvement of more than three anatomical regions was associated with significantly poorer prognosis, underscoring the importance of spatial distribution patterns in LM patients. Survival analysis demonstrated that dual third-generation targeted therapy conferred significant survival advantages in NSCLC-LM patients, whereas bevacizumab showed only marginal improvement that did not reach statistical significance. Furthermore, intrathecal pemetrexed dosage variations did not exhibit a statistically significant impact on survival outcomes within the studied cohort. Cox regression analysis revealed that multi-regional involvement (>3 regions) substantially increased the risk of frontal infiltration, with temporal lobe involvement potentially indicating critical disease progression.

Our findings demonstrate that NSCLC-LM predominantly manifests as a mixed phenotype, characterized by coexisting nodular and linear patterns with extensive lesion distribution. Compared to the pure linear type, the mixed phenotype is associated not only with greater tumor burden and CSF dissemination density but also with more severe neurological manifestations, including multiple cranial neuropathies, altered consciousness, and severe cephalalgia, accompanied by accelerated disease progression (17–19). The inherent biological aggressiveness of NSCLC cells facilitates the formation of highly vascularized solid metastatic nodules (nodular type) on the leptomeningeal surface, while CSF-mediated dissemination leads to extensive leptomeningeal enhancement (linear type), collectively establishing the pathological foundation of the mixed phenotype (14). Together, these features underscore the severe clinical manifestations and significant neurological compromise in NSCLC-LM patients, supporting the implementation of more aggressive multimodal therapeutic strategies (20–22).

This study utilized the high anatomical resolution of black blood sequences to perform precise spatial mapping of lesion distribution, establishing a significant correlation between extensive lesion dissemination and poor prognosis, thereby constructing a prognostic spatial model for NSCLC-LM. The model suggests that extensive distribution corresponds to survival challenges: patients with multi-compartmental involvement (e.g., simultaneous supra- and infratentorial lesions, or concurrent cerebral convexity, skull base, tentorial notch, and spinal cord infiltration) demonstrate significantly worse outcomes compared to those with localized disease (23, 24). Extensive dissemination serves not only as a direct indicator of the aggressiveness but also substantially increases therapeutic complexity, as pharmacological agents often fail to achieve adequate coverage across widespread metastatic sites. This condition is frequently associated with critical complications, including obstructive hydrocephalus, secondary severe neurological deficits, and refractory epilepsy (25).

Notably, temporal lobe involvement was identified as a robust adverse prognostic marker in NSCLC-LM. Supporting this observation, a concept increasingly recognized in neuro-oncology, where anatomic localization has been shown to influence both molecular profiles and clinical manifestations, as seen in gliomas (26). The temporal lobe, integral to higher cognitive functions (memory, language, emotion), limbic system integration, and sensory processing, often exhibits severe pathological consequences when compromised. Lesions in this region are associated with intractable epileptic seizures, marked cognitive decline, severe psychiatric and behavioral disturbances, and potentially fatal autonomic dysfunction. Consequently, imaging evidence of temporal lobe lesions should be regarded as a critical indicator of disease severity (27, 28).

Multivariate Cox regression analysis of imaging characteristics in this study demonstrated that frontal lobe involvement and an increased lesion count were significantly associated with elevated risk of poor prognosis in NSCLC-LM patients. Previous investigation has demonstrated a significant correlation between regions of reduced CSF velocity and the deposition of tumor cells, thereby elucidating the observed predilection for tumor involvement in these specific anatomical areas (29).

The administration of double-dose third-generation targeted agents, including osimertinib or furmonertinib, confers substantial survival benefits in patients with NSCLC and LM. Pharmacological evidence suggests that higher-dose regimens enhance blood-brain barrier penetration, thereby improving therapeutic efficacy. For example, furmonertinib monotherapy at 160 mg daily has elicited partial responses in patients harboring EGFR kinase domain duplication mutations, with median overall survival (OS) exceeding 22 months (30). These observations underscore the pivotal role of high-dose third-generation targeted agents in prolonging survival in this patient population. In addition, bevacizumab, an anti-angiogenic monoclonal antibody, may confer modest survival benefits in NSCLC-LM, though current evidence lacks statistical significance. Retrospective analyses indicate that EGFR-mutant NSCLC-LM patients treated with EGFR-TKIs in combination with anti-angiogenic agents, including bevacizumab, exhibit improved survival outcomes. Notably, furmonertinib combined with bevacizumab has demonstrated potential as a salvage therapy following resistance to third-generation EGFR-TKIs (31). However, further validation in larger prospective cohorts is warranted to substantiate these findings. Moreover, intrathecal pemetrexed (IP) represents a localized therapeutic approach for NSCLC-LM, though dose adjustments have not been associated with significant survival differences in the studied cohort (32, 33). A single-arm phase II trial revealed that IP therapy provided clinical benefit only in a subset of patients, with no discernible survival advantage observed across varying dosages (34). Current evidence suggests that dose optimization alone may have limited impact on survival outcomes, highlighting the need for combinatorial strategies to enhance therapeutic efficacy.

Additionally, this study revealed that patients with involvement of more than three brain regions exhibited significantly worse prognosis (mOS: 14 months vs. 24 months, P = 0.016), underscoring the importance of lesion spatial distribution in survival outcomes. Subsequent Cox regression analysis identified positive temporal lobe involvement (aHR = 1.96, P = 0.029) as independent adverse prognostic factors. The preferential involvement of the temporal regions in NSCLC-LM patients may be attributed to CSF circulation dynamics and anatomical characteristics (35, 36). This mixed imaging classification not only reflects complex imaging manifestations but also emphasizes the high-risk involvement of critical anatomical regions, warranting close clinical monitoring (36).

Moreover, involvement of more than three brain regions often indicates extensive disruption of the subarachnoid barrier, facilitating tumor cell release into the CSF (37, 38). Previous research has shown that the positive rate of CSF cytology in patients with widespread dissemination can reach 70%, whereas limited metastasis may result in false negatives due to lower tumor burden (9). Thus, the number of involved brain regions serves not only as a prognostic indicator but also as a potential determinant of CSF testing sensitivity and reliability. Therefore, imaging evidence of temporal lobe lesions serves as a crucial indicator of critical neurological conditions (39–41). In the multivariate Cox regression model, temporal lobe involvement remained an independent adverse prognostic factor. The protective effect of ≤3 involved regions persisted, further confirming that extensive spatial dissemination (>3 regions) independently predicts worse survival outcomes.

This study has several limitations. First, as a single-center retrospective investigation with a limited sample size, it is susceptible to selection bias. The exclusion of purely nodular types and the imbalanced distribution between mixed and linear types may compromise statistical power and the generalizability of the conclusions. These aspects should be validated through larger sample sizes and multicenter prospective studies. Second, due to the relatively uniform treatment strategies (predominantly targeted therapy combined with intrathecal chemotherapy), we were unable to assess the impact of different treatment regimens on prognosis. Future studies should explore the prognostic implications of therapeutic heterogeneity. Third, as the study included only NSCLC patients, the applicability of our findings to LM originating from other tumor types requires further investigation.

## 5 Conclusion

This study shows that MRI-based assessment of enhancement patterns and spatial lesion distribution reveals a strong association between mixed-type morphology with extensive involvement (>3 brain regions) and poor prognosis. Notably, temporal lobe is more susceptible to involvement. Additionally temporal lobe involvement signifies significantly reduced median survival. These findings offer an objective imaging basis for timely treatment strategy adjustment of treatment plans in clinical practice.

## Data Availability

The raw data supporting the conclusions of this article will be made available by the authors, without undue reservation.

## Supplementary material

Supplementary Table 1 Median survival time grouped by imaging characteristics.

Supplementary Table 2 Median survival time grouped by temporal lobe involvement.

Supplementary Table 3 Median survival time grouped by number of affected regions.
