# Supplementary material for: MRI-based morphological and spatial characteristics of leptomeningeal metastasis: prognostic value in non-small cell lung cancer
Source: Front Oncol. 2026 Apr 10;16:1764407. doi: 10.3389/fonc.2026.1764407 (PMC13105979; doi:10.3389/fonc.2026.1764407)
Supplement: Supplementary Table 1 — Median survival time grouped by imaging characteristics. [file Table1.docx]

Supplementary Table 1 Median survival time grouped by imaging characteristics

| Variables | N | Events | Median (95%CI) | Rate/1000 (person-months) | *Logrank P value* |
| --- | --- | --- | --- | --- | --- |
|  |  |  |  |  |  |
| MRI |  |  |  |  | 0.203 |
| linear | 41 | 31 | 22.00 (17.00 - 31.00) | 1631.58 |  |
| mixed | 30 | 24 | 15.00 (10.00 - 33.00) | 2000.00 |  |
