# Supplementary material for: MRI-based morphological and spatial characteristics of leptomeningeal metastasis: prognostic value in non-small cell lung cancer
Source: Front Oncol. 2026 Apr 10;16:1764407. doi: 10.3389/fonc.2026.1764407 (PMC13105979; doi:10.3389/fonc.2026.1764407)
Supplement: Supplementary Table 2 — Median survival time grouped by temporal lobe involvement. [file Table2.docx]

Supplementary Table 2 Median survival time grouped by temporal lobe involvement

| Variables | N | Events | Median (95%CI) | Rate/1000 (person-months) | *Logrank P value* |
| --- | --- | --- | --- | --- | --- |
|  |  |  |  |  |  |
| Temporal |  |  |  |  | **0.024** |
| no | 25 | 15 | 28.00 (19.00 - NA) | 441.18 |  |
| yes | 46 | 40 | 16.00 (12.00 - 25.00) | 2105.26 |  |
