# Supplementary material for: MRI-based morphological and spatial characteristics of leptomeningeal metastasis: prognostic value in non-small cell lung cancer
Source: Front Oncol. 2026 Apr 10;16:1764407. doi: 10.3389/fonc.2026.1764407 (PMC13105979; doi:10.3389/fonc.2026.1764407)
Supplement: Supplementary Table 3 — Median survival time grouped by number of affected regions. [file Table3.docx]

Supplementary Table 3 Median survival time grouped by number of affected regions

| Variables | N | Events | Median (95%CI) | Rate/1000 (person-months) | *Logrank P value* |
| --- | --- | --- | --- | --- | --- |
|  |  |  |  |  |  |
| Regionsinvolved |  |  |  |  | **0.016** |
| ＞3 | 38 | 30 | 14.00 (10.00 - 25.00) | 2500.00 |  |
| ≤3 | 33 | 25 | 24.00 (19.00 - 40.00) | 1315.79 |  |
